# Activation of epigenetic regulator KDM6B by *Salmonella* Typhimurium enables chronic infections

**DOI:** 10.1080/19490976.2021.1986665

**Published:** 2021-10-25

**Authors:** Sarika Rana, Sonalika Maurya, Gayatree Mohapatra, Savita Singh, Rohan Babar, Hridya Chandrasekhar, Garima Chamoli, Deepak Rathore, Pallavi Kshetrapal, C. V. Srikanth

**Affiliations:** a Laboratory of Gut Infection and Inflammation Biology, Regional Centre for Biotechnology, Faridabad, India; b Manipal Academy of Higher Education, Manipal, Karnataka, India; c Maternal and Child Health, Translational Health Science and Technology Institute, Faridabad, India

**Keywords:** *Salmonella* Typhimurium, KDM6B, H3K27me3, chronic infection, PPARΔ, SPI1 effectors

## Abstract

Non-typhoidal *Salmonella* (NTS) infections result in self limiting gastroenteritis except in rare cases wherein manifestations of chronic infections can occur. Strategies employed by *Salmonella* to thrive in hostile environments of host during chronic infections are complex and multifaceted. In chronic state, a coordinated action of bacterial effectors allows reprogramming of macrophages to M2 subtype and thereby creating a permissible replicative niche. The mechanistic details of these processes are not fully known. In the current study we identified, histone H3-lysine 27 trimethylation (H3K27me3)-specific demethylase, KDM6B to be upregulated in both cell culture and in murine model of *Salmonella* infection. KDM6B recruitment upon infection exhibited an associated loss of overall H3K27me3 in host cells and was *Salmonella* SPI1 effectors coordinated. ChIP-qRT-PCR array analysis revealed several new gene promoter targets of KDM6B demethylase activity including PPARδ, a crucial regulator of fatty acid oxidation pathway and *Salmonella-*persistent infections. Furthermore, pharmacological inhibition of KDM6B demethylase activity with GSKJ4 in chronic *Salmonella* infection mice model led to a significant reduction in pathogen load and M2 macrophage polarization in peripheral lymphoid organs. The following work thus reveals *Salmonella* effector-mediated epigenetic reprogramming of macrophages responsible for its long-term survival and chronic carriage.

## Introduction

*Salmonella*, a gram negative enteric pathogen, with more than 2500 serovars is capable of infecting broad range of host. Non-Typhoidal *Salmonella* (NTS) such as *Salmonella* Typhimurium causes gastroenteritis, a localized form of enteric infection that is resolved in 5–7 days in healthy individuals.^1^ NTS contributes for 93 million cases and 155,000 deaths worldwide annually.^2^ In very rare occasions, 0.15% in adults and 3.9% in children, NTS causes chronic infections which are of clinical and epidemiological importance. Chronic *Salmonella* infections contribute to not only recurrent episodes of disease and dissemination but also rare outcomes such as gallbladder cancer (GBC), colon cancer and reactive arthritis.^3–7^ Thus, NTS poses a significant economic burden and public health concern in developing nations such as Indian subcontinent and sub-Saharan Africa where newer invasive strains of NTS are also being reported.^8^

*Salmonella* pathogenesis is mediated through a battery of effectors encoded by *Salmonella* Pathogenicity Island I (SPI1) and *Salmonella* Pathogenicity Island II (SPI 2). These effectors are paramount in the effective mediation of Salmonellosis, and are translocated into the host by a specialized needle like complex or translocon referred as Type 3 secretion system (T3SS). The effectors are essential to *Salmonella* entry, intracellular replication and modulation of transcriptional profile and cellular signaling of the host cell.^9–11^ Activation and silencing of the host genes is achieved by complex strategies that also rely on post translational modification (PTMs) of host transcription regulatory proteins and many more that are yet to be discovered.^12,13^

*Salmonella* strains capable of causing persistent infections are adapted to circumvent host immune mechanism to ultimately thrive in phagocytic cells specifically within the macrophages in the reticulo-endothelial system. Through reticulo-endothelial system *Salmonella* disseminates systemically to other regions such as gallbladder, spleen, liver and bone marrow, and thereby persist for longer duration leading to chronic infection and carriage.^14^ Upon bacterial infection, macrophages mostly display classically activated M1 (pro inflammatory) subtype in response to various pathogen-associated molecular patterns (PAMPs) and the presence of inflammatory cytokines such as IFN-γ or TNF-α. M1 subtypes are microbicidal in nature and help in pathogen clearance.^15^ *Salmonella* through its SPI2 effectors dampens M1 polarization and shift the cells toward M2 subtype (here after M2-macrophage) preferentially at later stages of infection.^16^ Multiple recent reports have shown that *SteE*, a *Salmonella* SPI2 effector to induce polarization of M2-macrophages by phosphorylation of STAT3 through its interaction with GSK3. This mechanism in turn governs the expression of M2-specific IL10 cytokine and its cell surface marker arginase through the activation of transcription factor STAT3.^17–19^ M2 subtype with its anti-inflammatory traits and its reliance on alternative carbon source (fatty acids) for metabolism provides a distinct niche for *Salmonella* growth and persistence. During *Salmonella* infection, activated fatty acid oxidation regulator PPARδ modulates this metabolic shift in macrophages and allows persistence.^20^ However, the exact molecular mechanism involved in PPARδ upregulation during *Salmonella* infection remains unexplored.

Patho-epigenetic changes introduced by bacterial infections provide an effective means of quick, extensive and characteristic modulation of the host transcriptional machinery by the pathogens.^21^ A concerted interplay of transcription factors and epigenetic modulators play a critical role in reprogramming host cell as seen in case of *Listeria*,^22,23^ *Shigella*,^24^ *Helicobacter*^25,26^ and *Mycobacterium*.^27,28^ Epigenetic regulators, including histone modifiers play a critical role in activation and silencing of several inflammatory pathways. Pathogenic bacteria and even commensals take the advantage of this to control gastrointestinal immunity.^29,30^ Surprisingly, modulation of host histone modifiers and chromatin remodeling has not been explored in the context of *Salmonella* Typhimurium infections. Alterations in histone methylations governed by KDM6A,^31^ KDM6B^32,33^ and EZH2^34,35^ have been linked to macrophage polarization in several diseases, but have not been explored in the context of *Salmonella* infection.

In the current study, we investigated this possibility to discover the involvement of KDM6B- mediated histone demethylation in *Salmonella* pathogenesis. We demonstrate that upon *Salmonella* infection KDM6B upregulation is followed by a concomitant decrease in overall H3K27me3 mark of host, which required *Salmonella* SPI1 effectors. The activation of KDM6B demethylase activity was found necessary for the intracellular survival of bacteria in macrophages specifically at the later stages of infection, owing to M2 subtype polarization. This phenomenon could be attributed to KDM6B-mediated remodeling of PPARδ promoter and thus its activation. We were thus able to establish that KDM6B demethylase activity during *Salmonella* infection was necessary for the induction of M2 type macrophage polarization and required for long-term chronic carriage of *Salmonella*.

## Results

### Alteration in expression of histone demethylase, KDM6B during Salmonella Typhimurium infection

**Figure 1.**
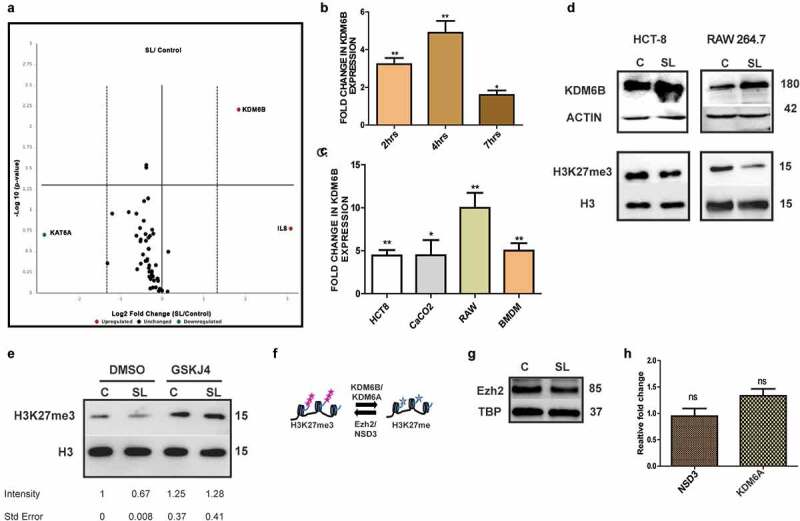
KDM6B recruitment and concomitant loss of H3K27me3 mark upon *Salmonella* Typhimurium infection [A.] Volacano plot showcasing fold change in expression profile of selected epigentic modifier in adenocarcinoma cell line HCT-8 post 4h of *Salmonella* Typhimurium (SL (SL1344 strain)) infection versus control cells.[B] Expression analysis of KDM6B at different time points post SL infection in HCT-8 cells and [C] in different cell lines post 4h of SL infection (p.i.) represented in fold change values by Quantitative Real Time PCR (qPCR) HPRT (Hypoxanthine-guanine phosphoribosyltransferase) was used for normalization [D.] Immunoblots showcasing KDM6B and respective Histone H3 lysine 27 trimethylation (H3K27me3 status at 4h p.i. in HCT-8 and RAW 264.7 cells.[E.] Immunoblot representing H3K27me3 upon SL infection upon treatment with KDM6B demethylase inhibitor GSKJ4 (30 µM) in RAW264.7 cells as indicated with respective fold intensity mentioned below. [F.] Pictorial representation of epigenetic modifiers (enzymes) involved in maintenance of H3K27me3 mark in cells. Immunoblot represeting expression levels of H3K27me3 methyltransferase Ezh2 [G] and NSD3 [H] using qRT-PCR at 4h post SL infection. KDM6A expression levels at 4h post SL infection analyzed using qRT-PCR (HPRT was used for normalization) [H]. TBP, Actin, H3 were used as loading control as indicated. Statistical significance was analyzed using unpaired t-test (’**’ *p*-value <.01, ‘*’ *p*- value <.05)

To study whether host epigenetic modulator(s) have any role to play in *Salmonella* infection, expression analysis was carried out using Qiagen-customized qRT-PCR array (RT-PCR array), which included several epigenetic modifier genes and control as shown in Tables 1 and 2. Human colonic epithelial cells (HCT-8 cell line) were either left untreated (control) or infected with wild-type *Salmonella* strain SL1344. In all the experiments involving cultured cells, *Salmonella* infection was followed by gentamicin treatment as done in gentamicin protection assay (GPA), unless indicated otherwise. Total RNA was isolated 4 h post infection (4 h p.i.) from these cells and used for RT-PCR array analysis. In infected samples, the results revealed an increase in expression of inflammatory chemokine IL-8, included in the array as a positive control for infection. Among the epigenetic modifier genes that were screened for, only KDM6B expression was found to be upregulated (>3 fold) in response to *Salmonella* infection (Figure 1a). KAT6A, a lysine acetyl transferase also seen to undergo expression alteration during *Salmonella* infection in our screen but the alteration was not statistically significant. Hence, in the current work we focused on KDM6B and its role in *Salmonella* pathogenesis. KDM6B is a histone demethylase belonging to JmjC domain containing family of histone demethylases known to specifically remove methyl group from trimethylated H3K27 (H3K27me3) at gene promoters leading to their activation.^36,37^ Expression analysis at different time intervals post *Salmonella* infection in HCT-8 cells showed a statistically significant increase in KDM6B expression with maximal expression at 4 h p.i. (Figure 1b). KDM6B upregulation, at 4 h post *Salmonella* infection, was also seen in intestinal epithelial cell line CaCo_2_, murine macrophage cell line RAW264.7 and primary bone marrow derived macrophages (BMDMs). Hence, these data indicated a conserved mechanism across different cell types in response to *Salmonella* infection (Figure 1c). Increased expression of KDM6B upon *Salmonella* infection was not dependent on live bacteria, as even heat killed *Salmonella* (HKS) was capable of triggering similar response suggesting a PAMP-mediated mechanism (Figure S1A). Furthermore, the upregulation of KDM6B in HCT-8 and RAW 264.7 cells was accompanied with a decreased global H3K27me3 in the infected, but not in untreated cells (Figure 1d). This phenomenon was also found to be conserved in BMDMs (Figure S1B). To further corroborate that decreased H3K27me3 mark was due to KDM6B demethylase activity, GSKJ4, a known pharmacological inhibitor of KDM6B demethylase activity, was used along with *Salmonella* infection.^38^ GSKJ4 treatment abrogated *Salmonella* infection dependent H3K27me3 demethylation (Figure 1e). To negate the role of other enzymes (Ezh2, NSD3 and KDM6A) known to contribute to genomic H3K27me3 mark (Figure 1f), expression analysis was carried out at 4 h p.i. Ezh2 and NSD3 are histone methylase that add methyl group to 27 lysine of Histone 3, whereas KDM6A is the other demethylase responsible for demethylation of H3K27me3. The expression of the above three genes remained unchanged during infection (Figure 1g-h). Together these data led us to conclude that *Salmonella* infection results in increased expression of KDM6B and a concomitant lowering of H3K27me3 mark in the host.

**Table 1.** Gene list for custom qPCR array

Sr. No.	Gene Name	NCBI Reference Sequence ID
1	PHF1	NM_002636
2	CBX1	NM_006807
3	INO80	NM_017553
4	CTBP1	NM_001328
5	SUZ12	NM_015355
6	MBD3	NM_003926
7	CBX7	NM_175709
8	ING4	NM_016162
9	EZH2	NM_004456
10	SPEN	NM_015001
11	ARID1A	NM_006015
12	ING2	NM_001564
13	MTA2	NM_004739
14	CBX8	NM_020649
15	BRD7	NM_013263
16	CHD8	NM_020920
17	PCGF2	NM_007144
18	EED	NM_003797
19	TRIM27	NM_006510
20	CBX4	NM_003655
21	BAZ2A	NM_013449
22	CBX3	NM_007276
23	ATF2	NM_001880
24	HAT1	NM_003642
25	KDM4A	NM_014663
26	CIITA	NM_000246
27	KAT2A	NM_021078
28	NCOA3	NM_181659
29	CARM1	NM_199141
30	PAK1	NM_002576
31	SETD6	NM_024860
32	KAT6A	NM_006766
33	HDAC6	NM_006044
34	HDAC10	NM_032019
35	EHMT2	NM_006709
36	KMT2C	NM_170606
37	AURKA	NM_003600
38	SETD1A	NM_014712
39	HDAC8	NM_018486
40	SMYD3	NM_022743
41	SUV39H1	NM_003173
42	DZIP3	NM_014648
43	USP21	NM_012475
44	KDM6B	NM_001080424
45	RPS6KA5	NM_004755
46	HDAC4	NM_006037
47	HDAC5	NM_005474
48	HDAC7	NM_001098416
49	IL8	NM_000584
50	UBE2I	NM_003345
51	ACTB	NM_001101
52	B2M	NM_004048
53	GAPDH	NM_002046
54	HPRT1	NM_000194
55	RPLP0	NM_001002
56	Human Genomic DNA Contamination	SA_00105
57	Reverse Transcription Control	SA_00104
58	Reverse Transcription Control	SA_00104
59	Reverse Transcription Control	SA_00104
60	Positive PCR Control	SA_00103
61	Positive PCR Control	SA_00103
62	Positive PCR Control	SA_00103

**Table 2.** List of primer sequence used

Sr.No.	Gene name	Primer Sequence 5ʹ-3’
Human Primer Sequence:		
1.	KDM6B Forward	GCACTACTGGGAGACCATCA
2.	KDM6B Reverse	ACCAGGAACCCGTCAAGTAG
3.	HPRT Forward	GCTATAAATTCTTTGCTGACCTGCTG
4.	HPRT Reverse	AATTAACTTTTATGTCCCCTGTTGACTGG
5.	KDM6A Forward	TACAGGCTCAGTTGTGTAACCT
6.	KDM6A Reverse	CTGCGGGAATTGGTAGGCTC
7.	NSD3 Forward	GTGTTAAGTTTCAGGTTGGCG
8.	NSD3 Reverse	CTGGACATGATATTCTCGGGC
9.	APC Forward	CATGATGCTGAGCGGCAGA
10.	APC Reverse	GCTGTTTCATGGTCCATTCGTG
11.	DAAM1 Forward	TCACCCAGAAATCACGTATCG
12.	DAAM1 Reverse	TCTGTGTTTGTCTGTGAGGTC
13.	FZD4 Forward	TACCTCACAAAACCCCCATCC
14.	FZD4 Reverse	GGCTGTATAAGCCAGCATCAT
15.	PPARδ Forward	GTCACACAACGCTATCCGTTT
16.	PPARδ Reverse	AGGCATTGTAGATGTGCTTGG
17.	WNT3 Forward	GGAGAAGCGGAAGGAAAAATG
18.	WNT3 Reverse	GCACGTCGTAGATGCGAATACA
19.	EP300 Forward	GACCAGACTACAGAAGCAGAAC
20.	EP300 Reverse	CACGGATCATACTTGGGTCAG
21.	CSNK1D Forward	CATCCCCTATCGTGAGAACAAG
22.	CSNK1D Reverse	AGCCCAGGTTGAAGTACATTAG
**Mouse Primer Sequence:**		
1.	HPRT Forward	GCTGGTGAAAAGGACCTCT
2.	HPRT Reverse	CACAGGACTAGAACACCTGC
3.	KDM6B Forward	TCGCTAAATACGCACAGTACC
4.	KDM6B Reverse	GGTTCCTGTAGTGCTGTCTG
5.	PPARδ Forward	CGGGCTCTAGAATTCCATCTG
6.	PPARδ Reverse	AGGTCTCACTCTCCGTCTTC
7.	IL1β Forward	CAGGATGAGGACATGAGCACC
8.	IL1β Reverse	CTCTGCAGACTCAAACTCCAC
9.	IFNγ Forward	TCTTGGCTTTGCAGCTCTTC
10.	IFNγ Reverse	TGTTGCTGATGGCCTGATTG
11.	ARG Forward	GGAATCTGCATGGGCAACCTGTGT
12.	ARG Reverse	AGGGTCTACGTCTCGCAAGCCA
13.	YM1 Forward	TCTGGTGAAGGAAATGCGTAAA
14.	YM1 Reverse	GCAGCCTTGGAATGTCTTTCTC
15.	IL10 Forward	TGCACTACCAAAGCCACAAG
16.	IL10 Reverse	TCAGTAAGAGCAGGCAGCAT
17.	IL4RA Forward	AGAGAATGTTAGTGTCAGTGTGG
18.	IL4RA Reverse	ACATGCTCAGGTCCTCTTTG
19.	ADRP Forward	AACAAAAGAGCCAGGAGACC
20.	ADRP Reverse	CCACGAGACATAGAGCTTATCC
21.	CPT1A Forward	ATCACCCCAACCCATATTCAG
22.	CPT1A Reverse	TGCGGGAAGTATTGAAGAGTC
23.	UCP2 Forward	TCCTGAAAGCCAACCTCATG
24.	UCP2 Reverse	GGCAGAGTTCATGTATCTCGTC
25.	ACADM Forward	CCGGAACACTTACTATGCCTC
26.	ACADM Reverse	TGTTGAATCCATAGCCTCCG
**ChIP qPCR Primer Sequence**		
27.	COX2_P Forward	AAGGGGAGAGGAGGGAAAAATTTGTG
28.	COX2_P Reverse	GAGGCGCTGCTGAGGAGTTCCTG
29.	GAPDH_P Forward	CTGCAGTACTGTGGGGAGGT
30.	GAPDH_P Reverse	CAAAGGCGGAGTTACCAGAG
31.	PPARδ _P Forward	TTTGACCAGCCCTAACTTCTC
32.	PPARδ_P Reverse	CCCCACAACTAAGCACTCG
**Primers for generation of *SteE* Knockout**		
33.	*SteE*KO1	GATCATACTTGTGGTTTTCCTTAGGAGGTAACATGTTTACAGTGTAGGCTGGAGCTGCTT
34.	*SteE*KO2	CTATTCGGCGCAGCTATTTATAACGCTTTGTTTTATTCAGTCATATGAATATCCTCCTTAG
35.	*SteE*1	AATTTAAGCTTAATGTTTACAATTAATAGTACTAACAGG
36.	*SteE*2	ATATGAATTCTCTTCATCCGGGAAAACC
37.	Cat1	TTATACGCAAGGCGACAAGG
38.	Cat2	GATCTTCCGTCACAGGTAGG

### KDM6B is recruited to promoters of WNT pathway genes, triggering transcriptional activation during Salmonella infection

**Figure 2.**
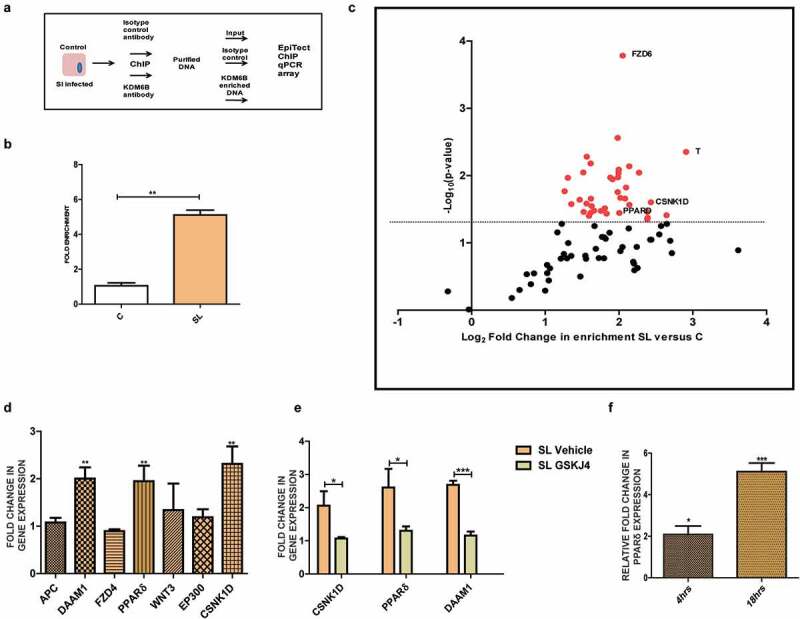
KDM6B binds to several WNT pathway gene promoters upon *Salmonella* infection: [A] Schematic representation of procedure employed for EpiTectChIP qPCR array. [B] Analysis of KDM6B binding at promoter of Cox2 gene in HCT-8 cells 4h p.i. of SL using ChIP. [C] Volcano plot showing KDM6B fold enrichment at several WNT pathway gene promoter post 4 h SL infection versus control in HCT-8 Cells using EpiTect qPCR array (Red dots are indicative of genes displaying *p*-value <.05; data is representative of two independent experiments). [D] Expression analysis of identified KDM6B target genes by above ChIP qPCR array at 4 h post SL infection in HCT-8 versus control cells. [E] Expression analysis of identified KDM6B target genes expression upon SL infection in the presence of KDM6B demethylase inhibitor GSKJ4 (30 µM). [F] Fold change in PPARδ expression at RNA level in RAW264.7 macrophages 4h and 18h p.i. w.r.t to control.(HPRT was used for normalization). Statistical significance was analyzed using unpaired t-test. (‘***’ *p*-value <.001;’**’ *p*-value <.01; ‘*’ *p*- value <.05)

The loss of gene repressive mark (H3K27me3) is indicative of transcriptional activation of target genes. To identify such target genes of KDM6B upon *Salmonella* infection, we carried out Chromatin immunoprecipitation coupled with RT-PCR array (hereafter ChIP-qPCR array; Figure 2a). The oligos used in the qRT-PCR corresponded to regions near TSS (transcription start site) of the gene promoters (Qiagen, GH-043A). Input DNA along with immuno-precipitated DNA with KDM6B or IgG antibody (negative control) from control and *Salmonella*-infected cells post 4 h was first analyzed using oligos specific to promoter of COX2, a known target gene of KDM6B.^39^ Compared to untreated cells, in *Salmonella* infected cells an increased binding activity of KDM6B was observed at COX2 promoter (Figure 2b).

Purified samples from the above experiment (input DNA, immuno-precipitated and negative control DNA) were subjected to quantitative real-time PCR array for detection of a range of gene promoters along with relevant control loci promoters. The data obtained was analyzed and a volcano plot was generated (Figure 2c), in which X axis indicate fold enrichment of KDM6B binding at the target gene promoter upon *Salmonella* infection in comparison to control sample. The Y axis reveals *P*-value or the significance of the result. Each dot on the graph represents data from a single promoter of tested genes. Notably, KDM6B displayed significant binding to promoters of several genes such as APC, DAAM1, PPARδ, WNT3, EP300 and CSNK1D (Figure 2c). After identification of promoters bound by KDM6B, it was important to examine if expression of these genes was actually modulated by KDM6B. Using qRT-PCR the expression of some of the KDM6B bound target genes was probed at 4 h p.i. DAAM1, a regulator of cytoskeletal reorganization, PPARδ, a fatty acid oxidation regulator and CSNK1D, a member of casein kinase gene family were found to be up-regulated upon *Salmonella* infection (Figure 2d). We next investigated whether the upregulation of these genes relied upon KDM6B histone demethylase activity by treating cells with GSKJ4. As anticipated, treatment of the cells with GSKJ4 and *Salmonella* infection negated the activation of these genes, indicating that their expression relied on KDM6B demethylase activity (Figure 2e). Among the identified genes, PPARδ was found to undergo a significant upregulation at later stages of infection in RAW264.7 macrophages (figure 2f). PPARδ, has a crucial role in fatty acid metabolism pathways required for macrophage biology as well as *Salmonella* persistence, and therefore was probed further.

### KDM6B-mediated alteration of H3K27me3 during infection is Salmonella effector driven

**Figure 3.**
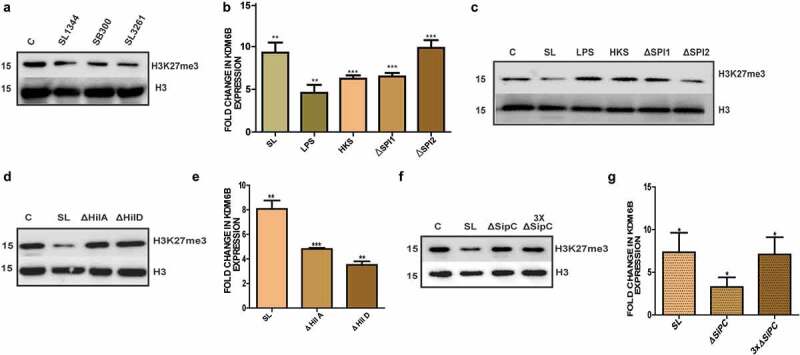
KDM6B demethylase activity dependent loss of H3K27me3 upon *Salmonella* infection requires release of SPI1 effectors: [A] Immunoblot representing H3K27me3 levels in RAW264.7 macrophages 4h p.i. with different strains of SL. [B] Expression analysis of KDM6B at RNA analyzed by qPCR (HPRT was used for normalization) and [C] immunoblot representing corresponding H3K27me3 levels 4h post treatment with *Salmonella* LPS (100 ɳg/ml), heat killed *Salmonella* (HKS), SL and mutants of *Salmonella* SPI1 (∆SPI1) and SPI2 (∆SPI2) in RAW264.7 macrophages. [D] Immunoblot representing H3K27me3 levels at 4h p.i. with SL, SPI1 effector regulator mutants ΔHilA and ΔHilD. H3 was used as loading control. [E] Fold Change in KDM6B expression in RAW264.7 4h upon infection with SL, SPI1 effector regulator mutants ΔHilA and ΔHilD (HPRT was used for normalization). [F] Immunoblot representing H3K27me3 levels at 4h p.i. with SL, SPI1 translocon protein SipC mutant ΔSipC and 3XΔSipC (3 times of MOI used for SL and SipC). H3 was used as loading control. [G] Fold change in KDM6B expression in RAW264.7 4h upon infection with SL, SPI1 translocon protein SipC mutant ΔSipC and 3XΔSipC(3 times of MOI used for SL and SipC)(HPRT was used for normalization)Statistical significance was analyzed using Student unpaired t-test. (‘***’- *p*-value <.001; ‘**’- *p*-value <.01, ‘*’ *p*-value <.05, ns- not significant)

*Salmonella*-mediated increased expression of KDM6B and global decrease in H3K27me3 levels was synonymous on infection with other wild type (SB300) and attenuated *aroA Salmonella* mutant (SL3261) strain as can be seen in (Figures S1C and Fig 3A). This indicates a conserved KDM6B upregulation and concomitant loss of H3K27me3 in macrophages upon infection with other wild type *Salmonella* strain SB300 and attenuated *aroA* mutant. To examine the cause of *Salmonella-* mediated KDM6B upregulation and alteration of H3K27me3 mark, we tested various *Salmonella* mutants devoid of effector secretion and *Salmonella* derived lipopolysaccharide (LPS) in RAW264.7 macrophages. Cells were treated for 4 h with either wild-type *Salmonella, Salmonella* SPI1- deficient mutant (ΔSPI1), SPI2-deficient mutant (ΔSPI2), heat-killed *Salmonella* (HKS), or with *Salmonella*-derived LPS. Post infection KDM6B expression by qRT-PCR and H3K27me3 levels using immunoblots were analyzed. KDM6B mRNA expression increased in all the treatments in comparison to control group (Figure 3b), however surprisingly the H3K27me3 levels remained unchanged on exposure to LPS, HKS and ΔSPI1. Only infection with wild-type *Salmonella* and ΔSPI2 displayed decrease in H3K27me3 (Figure 3c), thus indicating involvement of SPI1 effectors in loss of H3k27me3 levels.

SPI1-mediated loss of H3K27me3 was further explored in cells infected with *Salmonella* mutants of *hilA* (*ΔhilA*) and *hilD* (*ΔhilD*), strains deficient for regulators of SPI1 effector protein expression.^40,41^ Infection with *ΔhilA* or *ΔhilD* did not show any decrease in H3K27me3 levels (Figure 3d) but showed increased expression of KDM6B compared with control group (Figure 3e). *sipC* protein which forms an important component of translocon of the T3SS and is required for an efficient SPI1 effector release was also investigated.^42^ In line with the above data, infection with a *Salmonella sipC* mutant (Δ*sipC*), also did not lead to H3K27me3 levels decrease (Figure 3f), in spite of increased expression of KDM6B (Figure 3g). To compensate for reduced bacterial entry, which is a common phenomenon in the case of *sipC* defective mutants, three times the m.o.i of Δ*sipC* was used to infect the macrophages (Figure 3f). Increased m.o.i. did not lead to a discernable loss of H3K27me3 levels. Furthermore, even a five-fold increase of Δ*sipC* m.o.i did not lead to any noticeable change in H3K27me3 mark (Figure S1D). Together these data differentiate KDM6B activation from its demethylase activity in the context of *Salmonella* infection, and reveal requirement of SPI1 effector(s) in this process.

### PPARδ induction during Salmonella infection is dependent upon KDM6B demethylase activity

**Figure 4.**
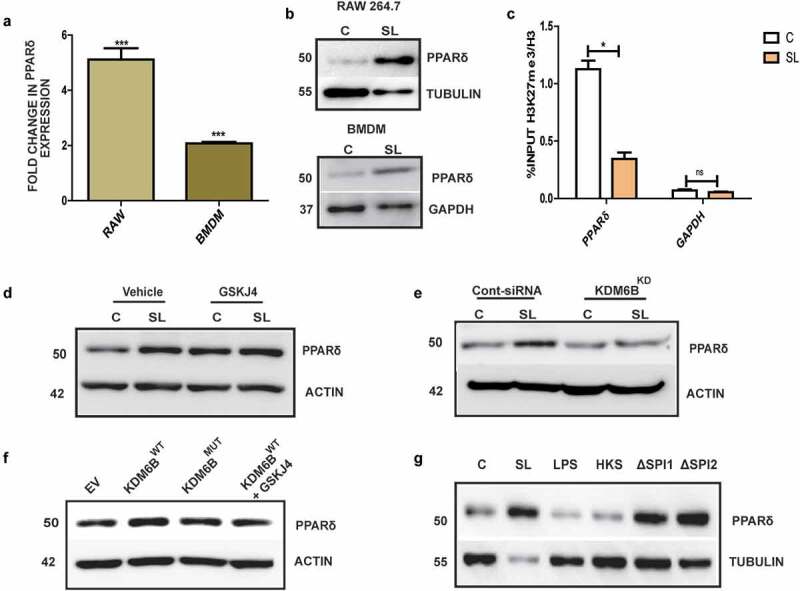
PPARδ activation upon *Salmonella* infection is mediated by KDM6B demethylase activity: PPARδ expression at RNA [A], and protein level [B] upon 18h p.i. in RAW264.7 macrophages and BMDMs.[C] % input enrichment of H3K27me3/H3 at PPARδ promoter upon 7 h p.i. using ChIP. Immunoblots indicating PPARδ protein expression at 18 hp.i. of *Salmonella* and treatment with GSKJ4 (30 µM) [D] or treatment with KDM6B siRNA [E]. [F] Immunoblot indicating PPARδ expression upon KDM6B wild type (KDM6B WT), KDM6B catalytic mutant (KDM6B MUT) upregulation in RAW264.7 cells. [G] Immunoblot showing PPARδ expression analysis post18h treatment with SL, LPS, HKS, and SPI1 or SPI2 mutant of *Salmonella* (Actin, Tubulin and Gapdh were used as loading control). Statistical significance was analyzed using unpaired student t-test. (‘***’p-value <.001, ‘*’ *p*-value <.05, ns- not significant)

Thus, far our data point toward an upregulation of KDM6B and a decrease in H3K27me3 mark during infection of host cells with *Salmonella* having functional SPI1 loci. These events lead to the recruitment of KDM6B to the WNT pathway genes such as PPARδ. We next proposed the role of PPARδ in *Salmonella* infection in greater detail. As seen in Figure 2c, KDM6B was enriched at PPARδ promoter leading to its transcriptional activation. In accordance to this, PPARδ was found to be upregulated at both RNA (Figure 4a) and protein level (Figure 4b) in RAW264.7 and BMDM cells upon 18 h p.i. with *Salmonella*. ChIP assay designed to specifically examine PPARδ promoter led us to find a decreased H3K27me3 mark at 7 h p.i. (Figure 4c). While, si-RNA-mediated KDM6B knockdown or GSKJ4 treatment of cells showed reduced or no up regulation of PPARδ upon 18 h post *Salmonella* infection (Figure 4d-e). GSKJ4-treated control cells showed an increase in PPARδ expression, which may be inadvertently due to activation of an alternate pathway owing to the drug activity. However, we do not see a further increase in PPARδ upon *Salmonella* infection along with drug treatment in comparison to drug-treated control cells. In addition, experimental perturbation through transfection of wild type (KDM6B^WT^) and catalytic mutant form of KDM6B (KDM6B^MUT^) demonstrated an increase in PPARδ expression only in the case of KDM6B^WT^, but not with KDM6B^MUT^ (Figures 4f and S1E). The above data thus suggest that KDM6B regulates PPARδ expression during *Salmonella* infection in macrophages. Furthermore, upon 18 h treatment of RAW264.7 cells with HKS, LPS, wild type *Salmonella* and *Salmonella* mutant SPI1 and SPI2 showed an increased PPARδ expression in the case of wild-type *Salmonella* and its SPI1 and SPI2 mutant. Thus, revealing that PPARδ expression depended on infection with live *Salmonella* (Figure 4g). We thus concluded that *Salmonella*-mediated recruitment of KDM6B results in the activation of PPARδ expression and this mechanism requires live *Salmonella*.

### KDM6B recruitment is imperative to infection in mesenteric lymph nodes of acute Salmonella colitis model

**Figure 5.**
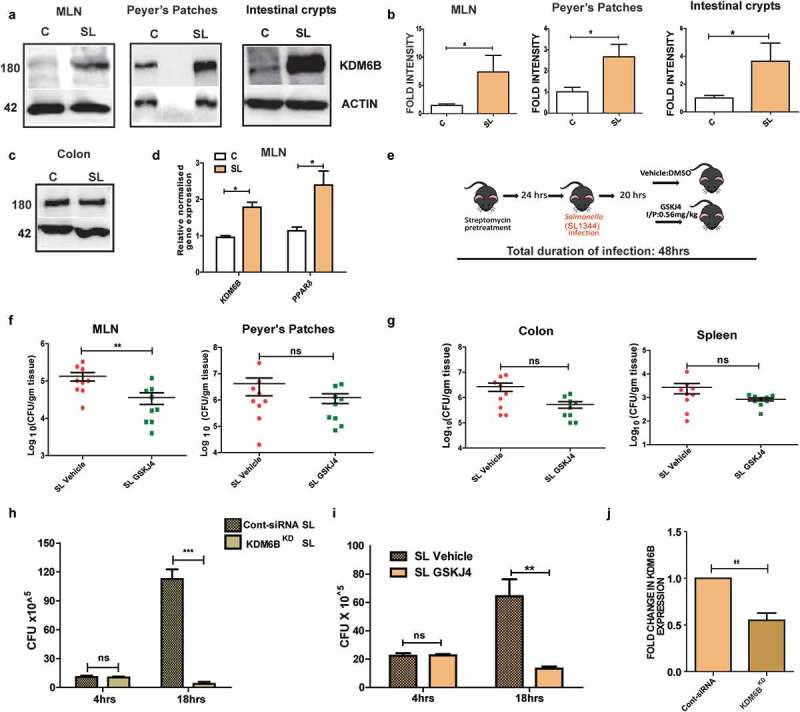
KDM6B demethylase activity is necessary for intracellular survival of *Salmonella* in murine MLNs: [A] Immunoblots showing increased expression of KDM6B mice in mesentric lymph nodes, Peyer’s patches and intestinal crypts in streptomycin pretreated mice model of SL 48h p.i. [B] Graphical representation of densitometry data for the above mentioned blots respectively (n = 6 mice per group). [C] Immunoblot showing KDM6B expression in colon at 48h p.i. of SL in C57BL/6 mice; Actin was used as loading control. [D] Fold change in KDM6B and PPARδ expression at RNA level in MLNs at 48h post SL infection in streptomycin pretreated mice colitis model using qPCR (n = 3 mice per group; HPRT was used for normalization). [E] Schematic representation of KDM6B demthylase inhibitor GSKJ4 mice *Salmonella* colitis model. [F-G] SL Colony forming units (CFU) in MLN, Peyer’s patches, colon and spleen in vehicle (DMSO) and GSKJ4 treated mice 48h p.i. (results are cumulative from 3 independent experiments). CFU of SL in RAW264.7 macrophages when treated with GSKJ4 (i), and upon knocking down of KDM6B by siRNA (KDM6B KD) (h) at 4h and 18h p.i. checked using GPAs. [J] KDM6B expression at RNA level in mouse KDM6B siRNA transfected RAW264.7 cells. Statistical significance was analyzed using unpaired t test and Mann-Whitney U Test. (’***’- *p*-value <.001; ‘**’- *p*-value <.01, ‘*’p-value <.05, ns-not significant)

To further understand the importance of KDM6B recruitment upon *Salmonella* infection in an *in vivo* context of infection, we studied its expression in Streptomycin-pretreated *Salmonella* colitis model (hereafter referred as SL Acute model).^43^ *Salmonella* wild type strain SL1344 was used to infect Streptomycin-pretreated C57BL/6 mice. At 48h p.i., when tissues from various organs of the mice were harvested and analyzed, KDM6B up-regulation was observed in specific regions namely intestinal crypts and immunologically privileged regions like Peyer’s patches and mesenteric lymph nodes (MLNs) (Figure 5a-b). On the other hand, no change in KDM6B expression was seen in colon (Figure 5c). Furthermore, PPARδ, the above-identified KDM6B target gene, was also found to be upregulated in MLNs of *Salmonella*-infected mice 48 h p.i. (Figure 5d).

To understand the importance of KDM6B in the intracellular survival of *Salmonella*, if any, the animals were treated with the inhibitor GSKJ4 or DMSO (vehicle) and the bacterial Colony Forming Unit (CFU) at 48 h p.i. in various organs was examined (Schematic of protocol followed is shown in Figure 5e). At 48 h p.i., *Salmonella* CFU in MLNs of GSKJ4-treated mice were found to be significantly lesser compared to the vehicle control (Figure 5f). Whereas, no significant change was observed in Peyer’s patches, colon and spleen of GSKJ4-treated and vehicle-treated *Salmonella* acute model (Figure 5f-g). In line with this, we did not observe any significant difference in colon length of *Salmonella*-infected mice compared to those that were *Salmonella*-infected and GSKJ4 treated (Figure S5B). *Salmonella* CFU was also found to be unaltered in the case of GSKJ4 or vehicle-treated HCT-8 cells at both early and late stage of infection indicating no role of KDM6B demethylase activity in *Salmonella* intracellular survival in epithelial cells (Figure S2E). The altered CFU could be either due to compromised dissemination of *Salmonella* from colon to MLNs, which is known to be executed by dendritic cells (DC).^44,45^ Therefore, *Salmonella* disseminating CX3CR1 positive dendritic cell populations (CD11c^+^ CX3CR1^+^) were analyzed using flow cytometry at 48 h p.i. in mesenteric lymph nodes of GSKJ4-treated and vehicle control mice. No significant change in the above cell population in drug-treated versus vehicle control was observed, thus suggesting inhibition of KDM6B demethylase activity did not affect *Salmonella* dissemination (Figure S2A-B). This could be further demonstrated by no change in CFU upon treatment with KDM6B inhibitor GSKJ4 in MLNs and spleen of mice at early stages (5 days) in chronic *Salmonella* mice model system (Figure S2C-D).

*Salmonella* thrives in host macrophages for long durations; therefore we tested possible involvement of KDM6B in *Salmonella* survival in macrophages. KDM6B was knocked down in RAW264.7 cells (KDM6B^KD^) and CFU post *Salmonella* infection at 4 h and 18 h was estimated using GPA and compared with mock treated RAW264.7 cells (Cont-siRNA). No change in CFU numbers was observed at 4 h p.i., but a significant decrease was observed at 18 h p.i. (Figure 5h). Similarly, treatment of cells with GSKJ4 drug led to a compromised *Salmonella* intracellular survival at 18 h p.i. (Figure 5i). GSKJ4 treatment of cells did not have any apoptotic affect varying from vehicle-treated cells as examined by PI staining (Fig S1F). KDM6B demethylase activity was imperative for long-term survival of *Salmonella* in macrophages for which *Salmonella* effector *SteE* has been shown to play critical role. We thus analyzed the effect of *Salmonella steE*mutant on KDM6B recruitment and its demethylase activity and found affect similar to wild type *Salmonella* (Figure S3D-E). Together these data indicate an imperative role of KDM6B in *Salmonella* intracellular survival in peripheral lymphoid organs.

### KDM6B is required for M2 type macrophage polarization during chronic Salmonella infection

*Salmonella* infection is known to modulate macrophage polarization to M2 for its sustained intracellular life, thus leading to persistence.^14^ PPARδ has been shown to play a critical role in this by modulating metabolic environment of the macrophages and thus polarization toward M2 subtypes expressing the (calcium-type lectin domain family 10 member A also known as CLEC10A) CD301 surface marker.^20^ Since our data revealed KDM6B to play a key role in PPARδ expression upon *Salmonella* infection and its intracellular survival, we hypothesized that this mechanism could be playing a role in *Salmonella* adaptation for a chronic infection in the host.

**Figure 6.**
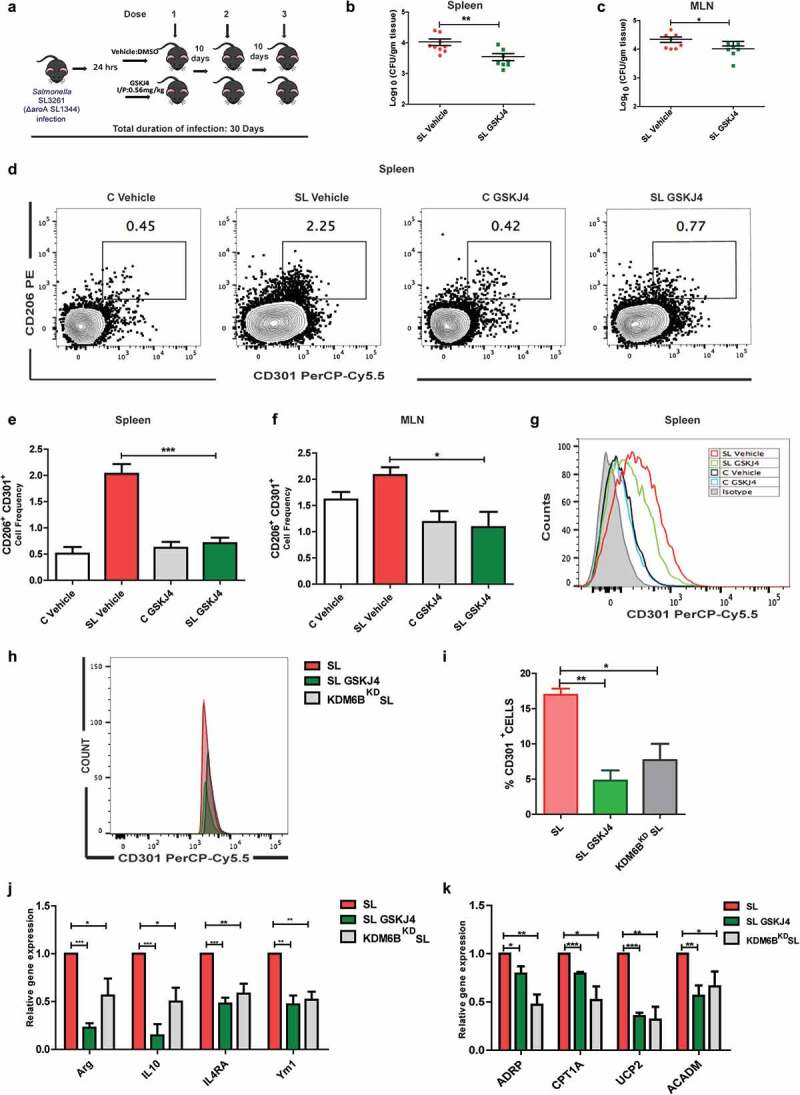
KDM6B demethylase activity is necessary for chronic *Salmonella* infection and M2 macrophage polarization. [A] Schematic representation of *Salmonella* chronic infection model (using attenuated aroA mutant SL3261 strain was used) along with GSKJ4 treatment. Panel [B] and [C] represent SL CFU obtained from spleen and MLN 30 days post chronic SL infection (dpi) in vehicle and GSKJ4 treated animals.[D] Representative of M2 macrophage population estimated using flow cytometry analysis of CD206+CD301+ double positive cell population on CD45.2+CD11b+Ly6G- cell population in spleen following 30 dpi. (Fig D is representative of FACS analysis of splenocytes of one of the mice from each group). CD206+CD301+ double positive cell frequency in spleen [E] and MLN [F] 30 days post chronic SL infection (dpi) in vehicle and GSKJ4 treated animals (% CD206+CD301+ population in CD45.2+CD11b+Ly6G-) (Gating strategy used is shown in Fig S4; n = 3 mice per group). Mean fluorescence intensity of CD301 expression on CD45.2+CD11b+Ly6G- population in spleen. [H] Flow cytometry analysis of CD301 population in GSKJ4 (5 µM) treated or KDM6B knock down BMDM at 24h p.i.[I] Graphical representation of CD301 population upon KDM6B knockdown or GSKJ4 treatment in *Salmonella* infected BMDMs in presence of IL4 (20 ng/ml). *Salmonella* infected or KDM6B perturbed BMDMs using GSKJ4 or KDM6B siRNA were anlyazed for M2 macrophage markers [J] and fatty oxidation pathway genes [K] using qRT-PCR array 24 h p.i. Statistical significance was analyzed using Mann Whitney U test (b-c), one way Anova analysis with post Tukey test (E,F,I) and student t- test (j-k). (‘***’- *p*-value <.001; ‘**’- *p*-value <.01, ‘*’ *p*-value <.05)

To test this hypothesis, the chronic infection model of *Salmonella* using attenuated *aroA* mutant strain of *Salmonella* (SL3261) to infect C57BL/6 mice was used. This allowed infected mice to survive for long durations needed for studying chronic infections. These animals were either treated with vehicle control (SL Vehicle) or GSKJ4 drug (SL GSKJ4) as shown in the schematic (Figure 6a). The animals were euthanized after 5 days and 30 days post infection (dpi) and MLN and spleen harvested were accessed for bacterial load. Even though, CFU in MLNs and spleen were not significantly altered by the presence of GSKJ4 at early stages of infection (Figure S2C-D). At 30 dpi a decreased bacterial burden in spleen and MLN was seen in mice treated with GSKJ4 (SL GSKJ4) in comparison to vehicle-treated mice (SL Vehicle) (Figure 6b-c). To address the reduced bacterial CFU in drug-treated mice, M2 type macrophage cell population expressing CD206 and CD301 cell surface markers (CD45.2^+^ CD11b^+^ Ly6G^−^ CD206^+^ CD301^+^; details of gating strategy in Figure S4A) known to harbor *Salmonella*, were examined in spleen and MLN of vehicle and drug treated mice at 30 dpi. CD206^+^CD301^+^ double positive macrophage population at 30 dpi showed ~ 4-fold decrease in SL GSKJ4 mice compared to SL Vehicle in spleen (Figure 6d-e) and ~2 fold decrease in MLN (Figures 6f and S4B). Mean fluorescence of CD301 surface expression on CD45.2^+^ CD11b^+^ Ly6G^−^ was also found to be reduced in GSKJ4-treated *Salmonella*-infected mice (Figure 6g). However, no significant change in the overall numbers of spleenocytes and immune cells in MLNs (Figure S4C-D) was observed. The difference in CD301 subpopulation was highly significant in spleen which also showed a decreased gene expression of M2 macrophage markers arginase1 (ARG), YM1 and interleukin 10 (IL10) in SL GSKJ4 mice compared to SL Vehicle mice (Figure S4C). Furthermore, SL GSKJ4 mice exhibited reduced gene expression of KDM6B and its target gene PPARδ in comparison to SL DMSO mice, as shown in qRT-PCR analysis (Figure S4C). Together these data implied that *Salmonella*-mediated KDM6B upregulation and its demethylase activity was required for polarization of macrophages and thus establishing chronic *Salmonella* infection.

In order to substantiate this finding, we checked *Salmonella* infection-mediated M2 polarization in BMDMs. BMDM cells which were either treated with GSKJ4 drug (SL GSKJ4), control siRNA (SL) or KDM6B-specific siRNA (KDM6B^KD^ SL) were infected with *Salmonella* (SL1344) in the presence of IL4 for 24 h as described in previous *Salmonella*-infected macrophage polarization studies.^17,20^ The resulting cells were then FACS analyzed for CD301 cell surface marker. CD301^+^ cell population as indicative from Figure 6h-i was severely compromised when the BMDMs were treated with either KDM6B siRNA (KDM6B^KD^ SL) or the GSKJ4 drug (SL GSKJ4), showing more than 2-fold decrease in CD301 expressing cell population. This was further evaluated for other M2 macrophage markers using qRT-PCR analysis. Expression of M2 macrophage markers Ym1, Arginase1, IL4RA (IL-4 receptor alpha) and IL10 were found to be significantly compromised in BMDMs treated with either GSKJ4 or KDM6B siRNA 24 h p.i. in comparison to those only infected with *Salmonella* (Figure 6j). The cells were further analyzed for fatty acid oxidation gene expression to determine whether KDM6B perturbations also affect the macrophage metabolic environment during *Salmonella* infection. KDM6B perturbations by either GSKJ4 treatment or KDM6B siRNA both resulted in reduced expression of PPARδ target ADRP (Perilipin 2), fatty oxidation pathway gene CPT1A (Carnitine Palmitoyltransferase 1A), UCP2 (uncoupling protein 2) and ACADM (Acyl-CoA dehydrogenase medium chain) in comparison with *Salmonella*-infected BMDMs 24h p.i., indicating a compromised fatty acid oxidation in KDM6B perturbed macrophages (Figure 6k). Together these data led us to conclude that *Salmonella* engages KDM6B demethylase activity to induce epigenetic reprogramming of macrophages toward M2 polarization to establish a successful chronic infection.

## Discussion

The current study reveals a role for KDM6B, a host demethylase, in epigenetic remodeling of specific gene loci in host macrophages enabling establishment of chronic *Salmonella* infections. *Salmonella* is capable of instigating a plethora of transcriptional changes in the host system, which is imperative to its pathogenesis.^46–49^ In young children, older patients and immune compromised host *Salmonella* is capable of systemic spread and resulting chronic infections.^50,51^ An intriguing long standing question has been that how *Salmonella* can reprogram phagocytes to establish a niche for its intracellular survival particularly during chronic infections.

Epigenetic modulation by pathogens is a fast-emerging theme in host–pathogen crosstalk, although modulation of histone modifiers in case of *Salmonella*-host interaction was unknown until the current study. Lacunae in the field of *Salmonella* patho-epigenetics led us to investigate the possible role of epigenetic modulators and histone modifiers in *Salmonella* pathogenesis and inflammation.

KDM6B/ JMJD3 is a lysine-specific demethylase that specifically removes di and trimethylated histone-3 lysine 27 (H3K27me2/me3). In the current study, we demonstrated *Salmonella* Typhimurium infection trigger an upregulation of KDM6B, which is shown to be a PAMP-mediated effect. These results are in line with previous studies that have shown KDM6B upregulation to be a TLR-mediated effect in macrophages stimulated with LPS (TLR-4) or infected with *Mycobacterium* (TLR2).^32,52,53^ As seen in the case of T-Box family member-dependent gene expression, gene activation mediated by KDM6B are not always necessarily dependent on its demethylase activity.^54^ Notably, an earlier study revealed that KDM6B-mediated induction of LPS-induced genes in phagocytes is not affected by KDM6B deletion and the induction remain independent of H3K27me3 alteration.^32^

The observed KDM6B upregulation was followed with a decrease in the host H3K27me3 during *Salmonella* infection. However, unlike increased KDM6B expression, the associated decrease in H3K27me3 levels required live *Salmonella* along with functional SPI1 loci. These findings are in line with the notion that LPS and HKS may be enough to upregulate KDM6B, but not its demethylase activity.^32,33^ Although *Salmonella* entry into phagocytic cells do not require SPI1 loci, induction of SPI2 genes, which are needed for intracellular survival depend on processes that are mediated by SPI1 genes.^55^ Further, a complete SPI1 arsenal may be required for the dissemination of *Salmonella* to remote sites in host wherein the activation of KDM6B may play a critical role in pathogenesis.

Through its effector proteins *Salmonella* circumvents and modulates host immune system and survives inside phagocytic cells especially, alternatively activated macrophages M2 subtype.^14^ Interestingly, KDM6B is also shown to be implicit in M2 macrophage polarization in response to helminths infection or treatment with chitin. In the study, demethylase activity of KDM6B was found to be indispensable for M2 macrophage polarization but not for M1 polarization.^33^ *Salmonella* employs specialized bacterial effectors to dismantle the M1 macrophage programming, thereby to establish a more permissible environment i.e. M2-like polarization for intracellular persistence. *Salmonella* SPI2 effector *SteE* has been recently shown to play a critical role in establishing M2 like subtype by the recruitment of transcription factor STAT3 to the nucleus and thus activation of genes like IL10 and arginase.^17,18,56^ Data from our work reveal that KDM6B activation was not being modulated by *SteE*.

Our work also established PPARδ, fatty acid oxidation regulator, and several other genes to be a target of KDM6B. In murine model of *Salmonella*, an earlier study has shown that PPARδ is required for fine tuning the metabolic environment of macrophages enabling chronic infection.^20^ PPARδ deficient mice show lower CD301 M2 subtype cell population upon *Salmonella* infection and hence are unable to sustain persistent infection. In our study, infection induced KDM6B-mediated demethylation at the promoter of PPARδ, led to its activation. Both GSKJ4 treatment and RNAi mediated KDM6B silencing in our work indicated lowered PPARδ expression and reduced bacterial number at later stages of infection in our *in vitro* system. Macrophage polarization at late stages of infection upon GSKJ4 treatment and KDM6B RNAi-mediated gene silencing showed impaired M2 polarization in terms of reduced CD301 expressing population in primary BMDMs. Furthermore, KDM6B-perturbed BMDMs also showed impaired expression of other M2 marker genes and fatty acid oxidation pathway genes upon *Salmonella* infection, indicating compromised *Salmonella* infection-mediated M2 polarization and fatty acid oxidation pathway upon KDM6B inhibition. In addition, GSKJ4-mediated KDM6B inhibition in chronic *Salmonella* infection mice model also displayed a reduced CD301 expressing macrophage population in MLN and splenic tissues. This decrease in M2 like macrophage subtype was in accordance with reduced bacterial CFU in respective organs upon GSKJ4 treatment indicating importance of KDM6B demethylase activity in establishing chronic infection. Apart from PPARδ, the work identifies new targets of KDM6B demethylase activity in the host upon *Salmonella* infection such as CSNK1D and DAAM1. The roles of these genes have not been yet investigated in *Salmonella* pathogenesis and could thus open new avenues in the field.

In this work, we have looked at the role of KDM6B in establishing permissible niche in macrophages but its role in *Salmonella* pathogenesis could be extensive. A more in-depth analysis and study of differential histone H3K27me3 alterations at the genomic level in response to heat-killed *Salmonella*, wild-type *Salmonella*, SPI effector mutants of *Salmonella* and new emerging invasive strains of NTS could shed light to different pathogeneses of the same bacterium.^57^ A very recent study in *Streptococcus pneumoniae* has shown how noninvasive pneumococcal isolate 6B ST90 showed increased expression of KDM6B followed by remodeling of IL11 promoter by its demethylase activity. This KDM6B activation and subsequent IL11 expression was absent in its invasive disease causing serotype TIGR4 thus leading to differing pathogenesis of the two serotypes.^58^

Chronic *Salmonella* infection has also been implicated in cancer cell transformation in genetically predisposed conditions. Thus, associating chronic *Salmonella* infection with colorectal and gallbladder cancer.^4^ The exact mechanism and pathophysiology of these diseases is still largely underexplored. Whether *Salmonella* infections, particularly chronic infections, also drive cancerous transformations of host as a result of epigenetic modifications remains an unanswered question. A recent study, wherein PPARδ was expressed under the control of villin-gene promoter in mice, has shown spontaneous development of invasive gastric adenocarcinomas highlighting the propensity of PPARδ to be carcinogenic.^59^ However, the role of PPARδ in APC heterozygous mice for development of colorectal cancer is quite controversial.^60^ KDM6B upregulation in intestinal crypts and PPARδ being its target and other identified WNT pathway genes, as shown in the above work, makes an interesting area of study in understanding long-term consequences of chronic *Salmonella* infection. Overall, the current study connects histone modifications and chromatin remodeling in *Salmonella*-host crosstalk, which may be critical for addressing some of the underexplored aspects of *Salmonella* pathogenicity such as chronic infections and long-term consequences of *Salmonella* infections including GBC.

## Materials and methods

### Cell culture

Human adenocarcinoma Cell lines HCT-8, CaCo_2_ and macrophage cell line RAW264.7 were grown in Sigma RPMI media supplemented with 14 mM NaHCO_3_ (Sigma), 15 mM HEPES buffer (pH 7.4) (GIBCO), 1 mM sodium pyruvate (GIBCO), 40 mg/L penicillin(GIBCO), 90 mg/L streptomycin (GIBCO), and 10% fetal bovine serum(GIBCO). BMDM were prepared from C57BL/6 as previously described.^20^ In brief, bone marrow was aseptically flushed out from the femur of mice using DMEM (Sigma). Bone marrow thus obtained were first treated with RBC lysis buffer (Sigma) and then cultured and differentiated in DMEM supplemented with 10 mM HEPES, non-essential amino acids (GIBCO), 10% FBS and 20% L929 conditioned media. BMDM were grown in DMEM low glucose medium (1 g/L) supplemented with 5% FBS and 5% L929 conditioned media 24 h prior to infection. For treatment with LPS from *Salmonella* Typhimurium (Sigma) 100 ng/ml, GSKJ4 (Sigma) 30 µM was used and IL4 (20 ng/mL) (Peprotech) treatment was given in BMDM polarization experiments as described earlier.^17^ All cell cultures were incubated in humidified 37°C incubator with 5% CO_2_.

### Bacterial growth and in vitro infection

*Salmonella* Typhimurium strain SL1344 (obtained from Dr Beth McCormick, University of Massachusetts Medical School, MA) was used for infection purpose except in experimentswhere mutant SipC, HilA and HilD were used, in which case SB300 strain was used, as knockout were in SB300 background (obtained from Dr Mrutyunjay Suar, KIIT college of engineering, Odisha). Further, for chronic *Salmonella* infection in mice Δ*aroA* mutant strain of *Salmonella* SL3261 was used (provided by Dr Bobby Cherayil, Massachusetts general hospital).

*Salmonella steE* mutant was prepared following the protocol of Datsenko and Wanner wherein SL1344 was transformed with temperature-sensitive plasmid pKD46 encoding the Lambda Red recombinase by electroporation. pKD46 transformed SL1344 were further transformed with amplified PCR fragment from plasmid pKD3 as template using Primers SteE KO1 and SteE KO2. The PCR product contained chloramphenicol cassette flanked by *SteE* homologous regions and transformants were selected on LB agar plates (Luria–Bertani) with 10 µg/ml chloramphenicol at 37°C to eliminate pKD46. The transformants were confirmed for *SteE* disruption using primers specific for *SteE* (SteE1 and SteE2) and cholaramphenicol (Cat1 and Cat2) cassette amplification.^61^ Plasmids pKD46 and pKD3 were provided by Dr Bobby Cherayil, Massachusetts general hospital.

*Salmonella* and its various mutants were grown in aerobic conditions at 37°C for 8 h. The primary culture was then diluted 1:1000, followed by overnight stationary and hypoxic growth conditions at 37°C as described earlier.^13,62,63^ Heat killed *Salmonella* was prepared by boiling the culture at 95°C for 10 min. Cells were infected at MOI of 1:40 for 1 h at 37°C, followed by treatment with gentamicin (Thermo Fischer Scientific) (100ug/ml) containing media for 1 h. The cells were then kept in the media containing gentamicin (10 µg/ml) for the rest of the infection. The above protocol is referred to as Gentamicin Protection Assay (GPA). *Salmonella* infection in BMDMs was carried out at MOI of 1:10 as described above. Cells were lysed using PBS +.1% triton X-100 followed by serial dilution and plating on streptomycin (50 µg/mL) containing LB agar plate for bacterial burden assay. The plates were incubated in a 37 degree incubator and bacterial CFU were counted. All experiments were carried out according to Institutional bioethics committee guidelines.

### Transfection and knock-down experiments

RAW 264.7 cells (3 × 10^5^) were seeded onto 24-well plates one day prior to transfection to obtain 70–80% confluency. The next day cells were transfected with appropriate plasmids using Lipofectamine 2000 (Invitrogen) according to the manufacturer’s instructions. Briefly, 1 μg of plasmid (addgene-17440)^30^ and lipofectamine 2000 were separately diluted in Opti-MEM (In vitrogen, Carlsbad, CA) and incubated for 5 min. The two mixtures were then mixed and incubated for another 20 min at room temperature followed by addition to the cells kept in Opti-MEM media and incubated for 12 h in the incubator. 12 h post-incubation media was changed and fresh complete media without and with GSKJ4 (5 µM was added) and incubated for another 24 h before harvesting the cells for RNA and protein. For siRNA-mediated knockdown 25pmol of non targeting siRNA and KDM6B siRNA (ON-TARGETplus SMARTpool, Dharmacon, GE) was mixed with Opti-MEM for 5 min at RT. Separately, transfecting reagent Dharmafect (GE) was also mixed with Opti-MEM for 5 min at RT. The two mixtures were then mixed and incubated for 20 min at RT. The mixture was then mixed with RAW264.7 (3 × 10^5^) cells and BMDM (2 × 10^6^) cells and seeded onto 24-well and 6-well plate, respectively. Six hours post transfection media was changed to complete media in case of BMDM.

### Mice infections

C57BL/6 mice (6–8 weeks) were used throughout the study. For acute *Salmonella* colitis model, food and water was removed 4 h prior to infection followed by a treatment with 20 mg/Kg of streptomycin by oral gavage. *Salmonella* (SL1344) (CFU at 5x10^7^) was fed to mice using oral gavage the following day after 4h withdrawal of food and water. Mice were euthanized 48 h post *Salmonella* infection and various organs were harvested. For isolation of intestinal crypts from colon, Gentle Cell Dissociation Reagent (Stemcell^TM^ technologies) was used using manufacture’s prescribed protocol. Chronic infection of *Salmonella* in mice was carried out using *aroA* mutant attenuated *Salmonella* SL3261 strain (10^9^ CFU per mice) using oral gavage (food and water was not removed).^20^ For GSKJ4 *Salmonella* colitis model, GSKJ4 (.56 mg/kg) was given 20 h post *Salmonella* infection intraperitoneally.^64^ In case of GSKJ4 chronic infection model GSKJ4 was given in total of 3 doses 10 days apart for complete 30 days of *Salmonella* infection. For bacterial burden assay in the above-described mouse model systems, mice were euthanized post infection and indicated organs were harvested and homogenized in PBS (.1% triton X-100). The homogenized tissues were then serially diluted and plated onto MacConkey agar plates. The plates were incubated in a 37-degree incubator and bacterial CFU counted. All animal experiments were carried out in the Small Animal Facility of Regional Center for Biotechnology (RCB). Animal ethics proposal were approved by the RCB Institutional Animal Ethics Committee (approval no. IAEC/RCB/2018/044 and IAEC/RCB/2019/061).

### Immunoblotting

Cells were lysed in RIPA buffer (Sigma) supplemented with Protease Inhibitor Cocktail (G biosciences). SDS Laemelli buffer was then added to protein lysates followed by boiling at 95 C. Protein was quantified using CBX™ protein assay kit (G-Bioscience, USA). Prepared protein lysates were then separated using sodium dodecyl sulfate–polyacrylamide gel electrophoresis and transferred to nitrocellulose membrane (BioRad). Blots were probed with antibodies against KDM6B (Abcam-ab38113), Actin (CST-4970S), Tubulin (Sigma-T7816), PPARδ (Abcam-ab8937), Ezh2 (CST-5246S). In order to analyze the H3K27me3 mark histone protein were extracted using (.2 N HCL) acid extraction. Extracted histone proteins were then run on a SDS page gel and immunoblotted for H3K27me3 mark (CST-9733S) and H3 (Abcam-ab1791). Blots were developed using GE ImageQuant LAS4000 and densitometry analysis was done using the analysis software of the instrument.

### qRT-PCR and qRT-PCR array

RNA was isolated from the samples using Nucleospin RNA kit (Macherry-Nagel, Germany). 1 μg of isolated total RNA for each sample was used to synthesize c-DNA using Bioradi-Script cDNA synthesis Kit. Bio-Rad CFX 96™Real-Time Detection System was used for real-time PCR (qRT-PCR) using i-TaqSyber green (Bio-Rad, USA) according to manufacturer’s instruction. In case of qPCR array, 1 µg of isolated RNA was used to synthesize cDNA using RT^2^ first strand kit (QIAGEN, SA Biosciences, USA). The real-time PCR array was then performed using RT^2^Syber Green Master Mix (QIAGEN, SA Biosciences, USA) on 96 well plate of customized qRT-PCR array plate. The qRT-PCR reaction was performed according to the manufacturer’s protocol and Bio-Rad CFX 96™- Real Time Detection System (Bio-Rad, USA was used. The data thus obtained were then analyzed using RT^2^ Profiler™ PCR Array Data Analysis Web-based tool (QIAGEN, SA Biosciences, USA) having following URL: http://pcrdataanalysis.sabiosciences.com/pcr/arrayanalysis.php.

### ChIP and ChIPqRT-PCR array

The cells post infection were crosslinked with 1% formaldehyde (Sigma) at RT for 10 min followed by quenching with 125 mM glycine for 5 min. The cells were then washed with PBS and collected. Cells were then lysed using lysis buffer (10 mM HEPES-KOH (pH 7.9), 85 mM KCl (Sigma), 1 mM EDTA (Sigma),1% NP-40 (Sigma), protease inhibitor cocktail) followed by centrifugation at 2000rpm for 10 min at 4 C. The nuclei were then lysed using nucleus lysis buffer (50 mM Tris Cl (pH 8), 10 mM EDTA, 1% SDS, protease inhibitor cocktail) for 10 min on ice. The lysed samples were then sheared using a sonicator to obtain chromatin fragments of 200–500bp. Sheared chromatin was then used for immunoprecipitation using ChIP grade antibodies specific for KDM6B (ABCAM, ab38113), H3K27me3 mark (CST-9733S) and H3 (Abcam-ab1791), IgG control (ABCAM, ab171870) acting as negative control overnight at 4°C. The immunoprecipitated DNA was then pulled using DynaBeads^TM^protein G (*In vitro*gen) through incubation at 4°C for 3 h

The immunoprecipitated DNA and the input DNA was purified using DNA purifying slurry (Diagenode, C03080030). The purified DNA was used for performing qPCR. For ChIP qRT-PCR array the purified DNA from input sample and immunoprecipitated by KDM6B and IgG antibodies was mixed with RT2 SYBR Green qPCR Mastermix and added to Qiagen EpiTect ChIP qPCR array plate (Qiagen, GH-043A). The plates were then run on qPCR machine Bio-Rad CFX 96™. The data were collected, analyzed and plotted as volcano plot using Graph pad prism.

### Immuno-phenotyping and multicolor flow cytometry

MLN and spleen were aseptically harvested followed by preparation of a single cell suspension. The cells were passed through a 40 µM BD strainer. 1 million cells were taken and blocked using CD16/32 (Biolegend-101302) antibody for 10 min on ice. The cells were surface stained with fluorescently labeled antibodies for CD45.2 (Biolegend-109824), Ly6G (Biolegend-127614), CD11c (Biolegend-117311), CX3CR1 (Biolegend-149006), CD11b (Biolegend-101251), CD206 (141705), CD301 (Biolegend-145710). Fluorescently labeled cells were washed twice with PBS and acquired on BD FACS Canto II and BD FACS Verse (BD Biosciences, USA). The acquired data were then analyzed using FlowJo^TM^ 10 software.

### Statistics

All the results are expressed as the mean standard error from an individual experiment done in triplicate. Data were analyzed with one way ANOVA followed by Tukey’s posttest, standard unpaired two-tailed Student’s t test and the Mann–Whitney U-test was used where applicable, with *p* values of .05–.001 considered statistically significant. We evaluated the statistics with GraphPad PRISM.

## Supplementary Material

Supplemental Material
