# Supplementary material for: Activation of epigenetic regulator KDM6B by Salmonella Typhimurium enables chronic infections
Source: Gut Microbes. 2021 Oct 25;13(1):1986665. doi: 10.1080/19490976.2021.1986665 (PMC8555538; doi:10.1080/19490976.2021.1986665)
Supplement: Supplemental Material [file KGMI_A_1986665_SM3264.zip › Supplementary information/Supplementary figures caption.docx]

**Supplementary Figure S1:** [A] Fold change in KDM6B gene expression at RNA level post 4 hrs of treatment with SL and heat killed *Salmonella* (HKS). [B] Immunoblot representing loss of H3K27me3 level post 4 hrs of *Salmonella* infection in BMDM cells. [C] Fold change in KDM6B expression at RNA level in SL1344, SB300 and SL3261 infected RAW264.7 macrophages at 4hrs post infection. [D] Immunoblot representing H3K27me3 levels upon 4hrs of SL and 5x SipC (5 times of MOI used for SL) infection. [E] Fold change in KDM6B expression upon transfection of KDM6B and its catalytic mutant expressing vector w.r.t empty vector transfected cells. [F] Drug GSKJ4 (30µM) did not lead to significant cell death than the vehicle treatment of cells detected by PI staining of cells post 7 hrs of treatment. Statistical significance was analyzed using Student unpaired t-test. (‘***’- p-value<0.001; ‘**’- p-value<0.01, ‘*’ p-value <0.05, ns- not significant).

**Supplementary Figure S2: KDM6B demethylase activity inhibitor GSKJ4 does not affect *Salmonella* dissemination.** Flow cytometry analysis of *Salmonella* disseminating cell CD11c+ CX3CR1+ (Q2) dendritic cells in total immune cells obtained from mesentric lymph nodes, in vehicle and GSKJ4 *Salmonella* colitis model post 48hrs p.i. [A]. Graphical representation of CD11c+ CX3CR1+ cell frequency in mesentric lymph nodes showing no discernible difference between vehicle and GSKJ4 treated and Salmonella infected samples (n=5 mice per group) [B]. Bacterial CFU in MLNs [C] and spleen [D] of vehicle and GSKJ4 treated chronic *Salmonella* infection model 5 dpi (attenuated aroA mutant strain SL3261 was used) (n=6 mice per group). [E] Bacterial CFU in vehicle and GSKJ4 (30µM) treated adenocarcinoma cell line HCT-8 post 4hrs and 18hrs p.i.. analysed using GPA. Statistical significance was analyzed using one way Anova post Tukey test, Mann Whitney U test and unpaired t-test.(‘***’- p-value<0.001; ‘**’- p-value<0.01, ‘*’ p-value <0.05, ns- not significant).

**Supplementary Figure S3: *SteE* mutant of *Salmonella* is capable of mediating KDM6B recriutment and associated loss of H3K27me3.**Verification of *SteE* disruption in *SteE* knockout of SL1344 by PCR amplification for *SteE* in wildtype (SL) and *SteE* mutant (Δ*SteE*) [A] and presence of Cholramphenicol cassette in wildtype(SL) and *SteE* mutant (Δ*SteE*) by PCR [B].[C] Immunoblot representing p-Stat3-Tyr705 levels in uninfected, SL and *SteE* mutant infected RAW264.7 post 18 hrs of infection. [D] Fold Change in KDM6B expression levels in RAW264.7 cells post 4hrs of infection with wildtype SL and *SteE* mutant in comparison to uninfected cells (HPRT was used for normalisation). [E.] Immunoblot representing H3K27me3 levels inRAW264.7 cells post 4hrs of infection with wildtype SL and *SteE* mutant (H3 was used as loading control). Statistical significance was analyzed using Student t-test. (ns- not significant).

**Supplementary Figure S4: GSKJ4 mediates decrease in M2 macrophage marker expression during Chronic *Salmonella* infection** [A] Gating strategy for M2 macrophage (CD206+CD301+ double positive) population analysis obtained from mice Spleen and MLN using FMO (Fluorescence minus one). [B] Representative of M2 macrophage population estimated using flow cytometry analysis of CD206+CD301+ double positive cell population on CD45.2+CD11b+Ly6G- cell population in MLN following 30 dpi. Total spleenocytes in spleen [C] and immune cells in MLN [D] in control vehicle (C-Vehicle) and GSKJ4 (C-GSKJ4) treated mice post 30 days of chronic *Salmonella* infected mice in vehicle and GSKJ4 groups (SL Vehicle and SL GSKJ4 respectively). [E] Fold change in gene expression of M2 macrophage markers (Arginase, Ym1, IL10), KDM6B and its target PPARδ in spleens of vehicle and GSKJ4 treated mice 30 days post chronic *Salmonella* infection (n=3 mice per group). Statistical significance was analyzed using one way Anova post Tukey test and Student t-test. (‘**’- p-value<0.01, ‘*’p-value <0.05, ns-not significant)

**Fig S5: A.** Immunoblots representing KDM6B expression in RAW264.7 macrophages 4hrs (left panel) and 18hrs (right panel) post treatment with *Salmonella* (SL), Heat Killed *Salmonella* (HKS) and *Salmonella* LPS (100ŋg/ml). Actin was used as loading control. **B.** Representative image indicating colon length in control and *Salmonella* infected mice in presence and absence of KDM6B demethylase inhibitor GSKJ4 48 hrs p.i. in Streptomycin colitis mice model system. Right panel is a graph representing average colon length (n=2-3 mice per group).
