# Supplementary figures and images for: Activation of epigenetic regulator KDM6B by Salmonella Typhimurium enables chronic infections
Source: Gut Microbes. 2021 Oct 25;13(1):1986665. doi: 10.1080/19490976.2021.1986665 (PMC8555538; doi:10.1080/19490976.2021.1986665)

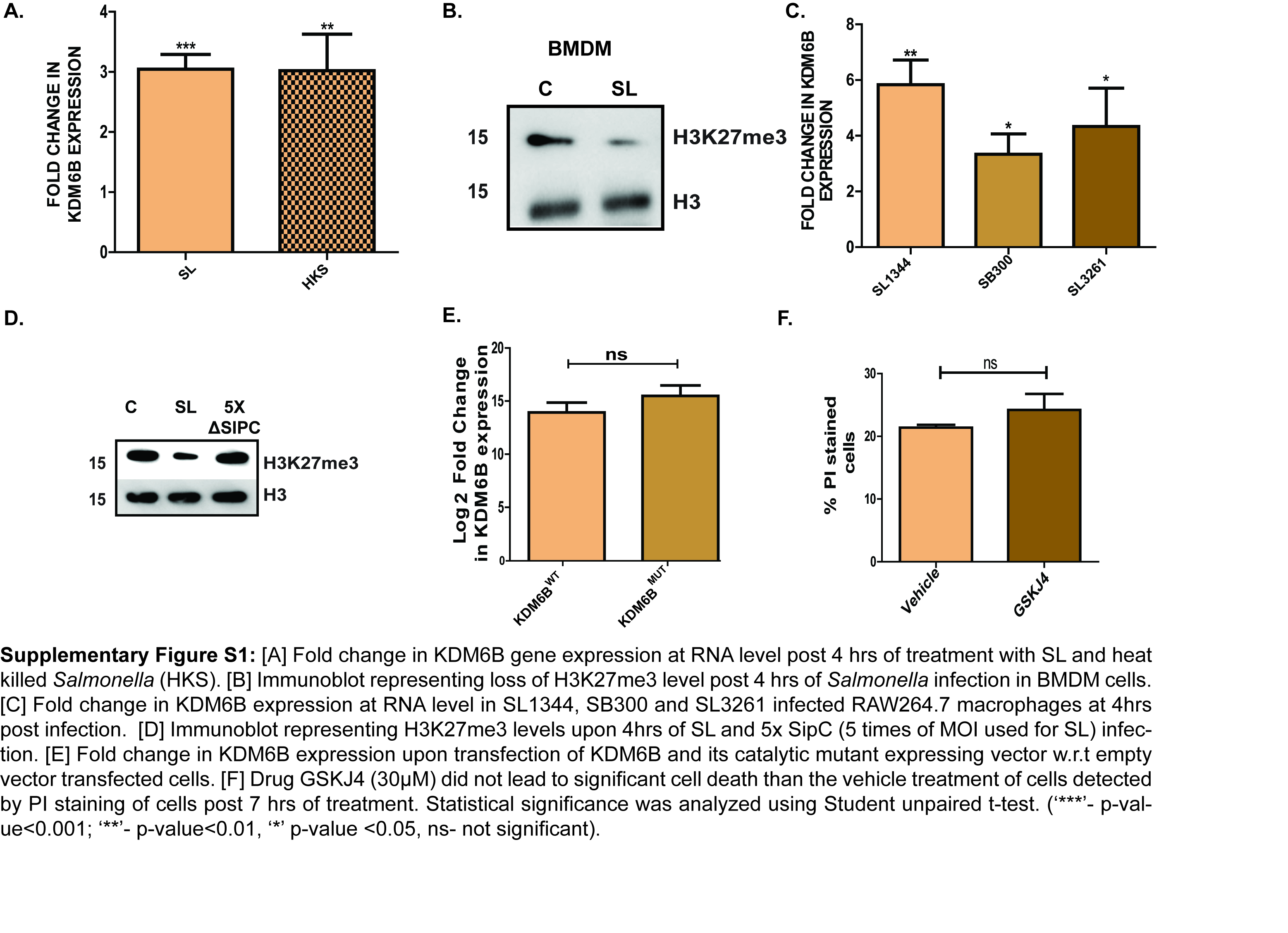

Supplement: Supplemental Material [file KGMI_A_1986665_SM3264.zip › Supplementary information/SUPPLEMENTARY FIGURE S1.tif]

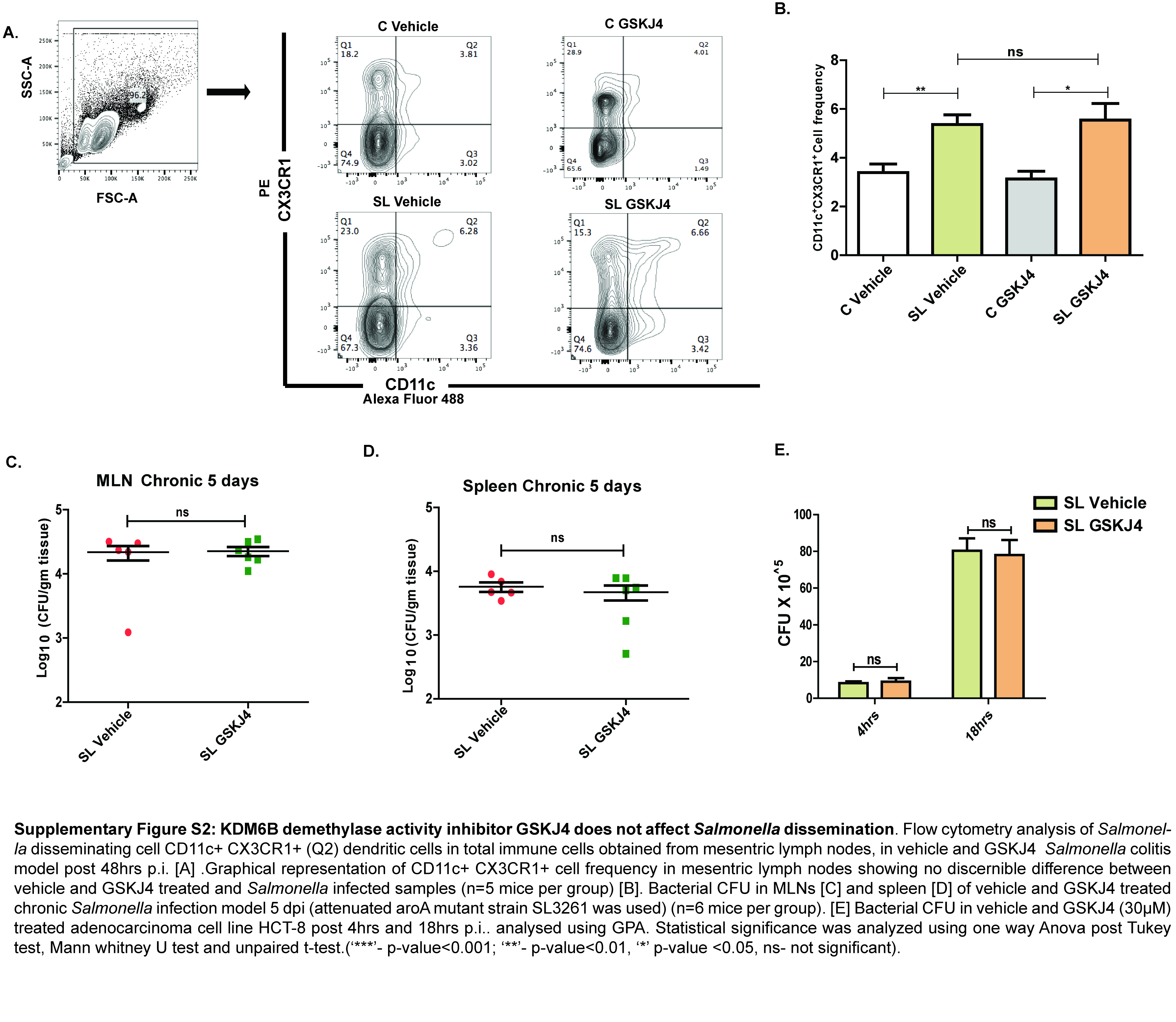

Supplement: Supplemental Material [file KGMI_A_1986665_SM3264.zip › Supplementary information/supplementary Figure S2.tif]

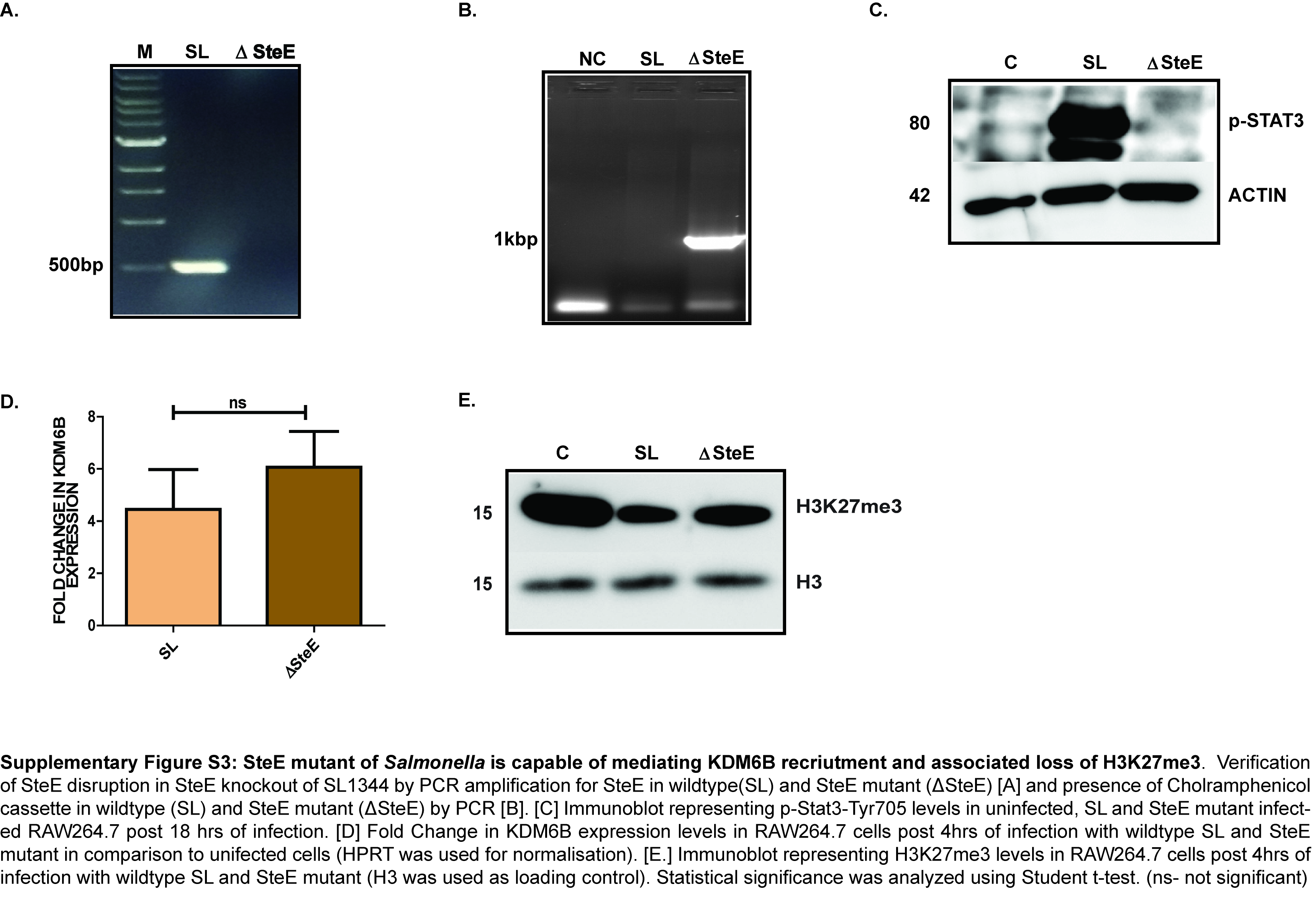

Supplement: Supplemental Material [file KGMI_A_1986665_SM3264.zip › Supplementary information/Supplementary Figure S3.tif]

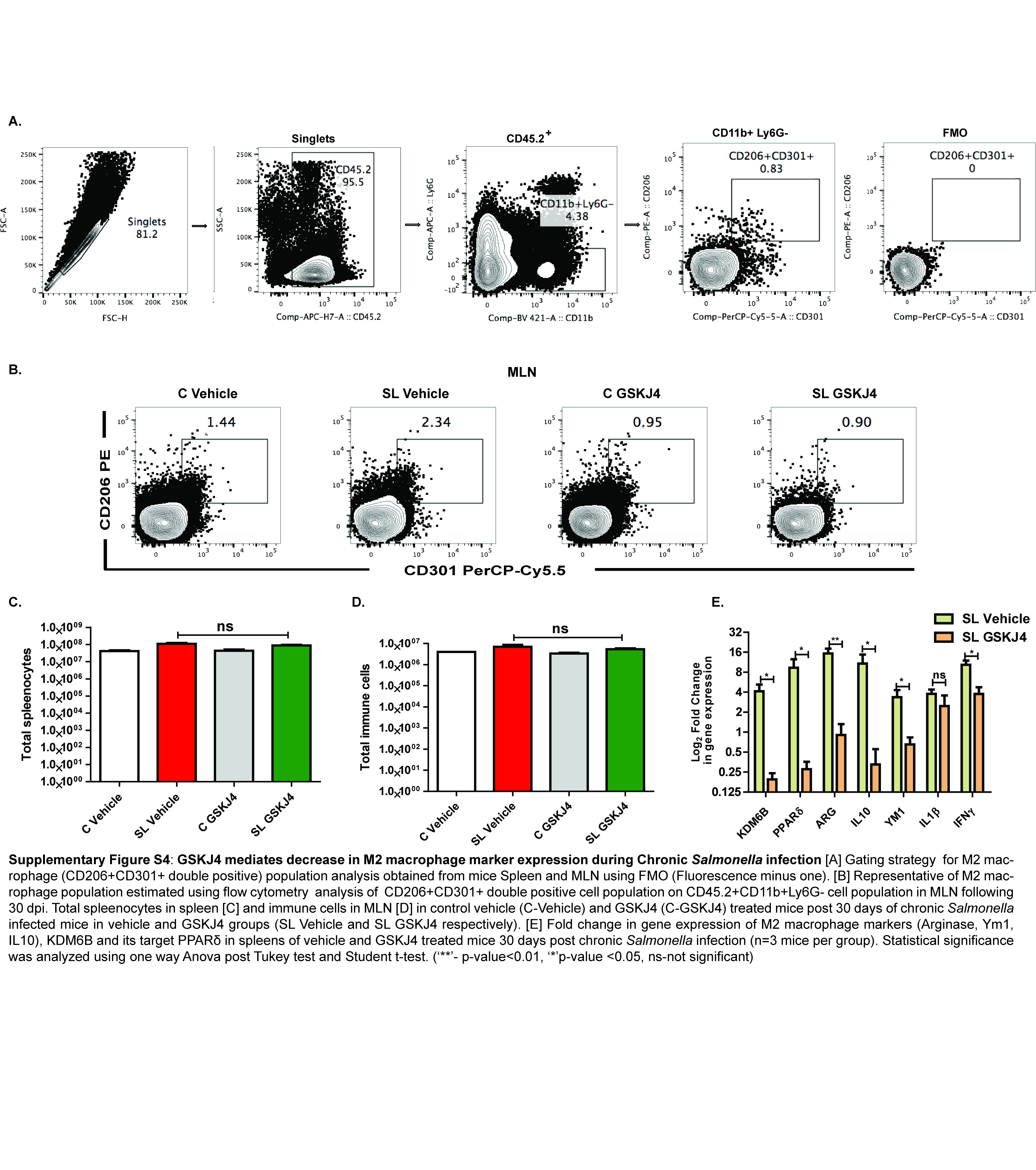

Supplement: Supplemental Material [file KGMI_A_1986665_SM3264.zip › Supplementary information/supplementary figure S4.tif]

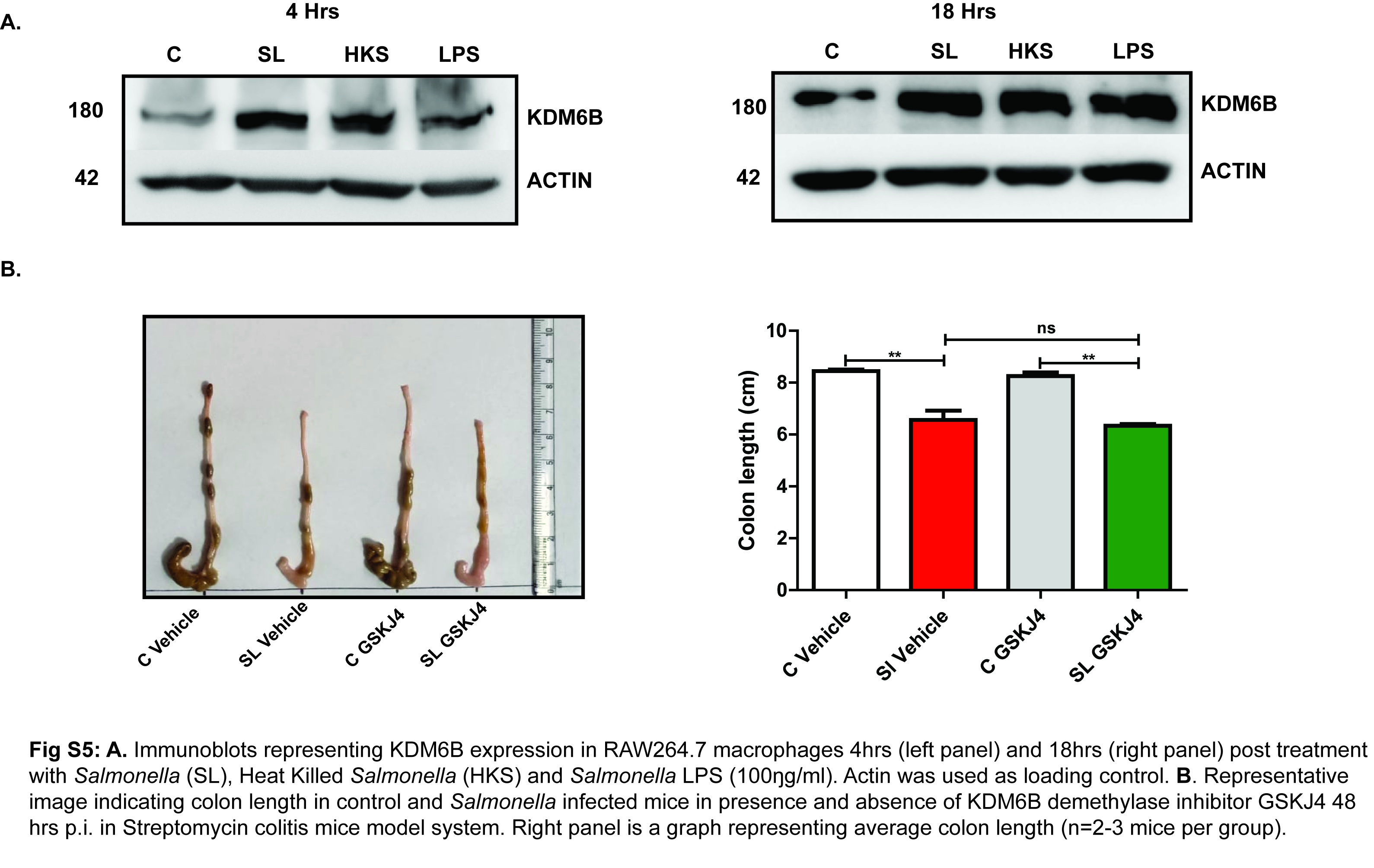

Supplement: Supplemental Material [file KGMI_A_1986665_SM3264.zip › Supplementary information/Supplementary Figure S5.tif]
